# Editorial I: So, who wins? It is still: NCDs 3; Infections 2

**DOI:** 10.4314/ahs.v22i3.1

**Published:** 2022-09

**Authors:** James K Tumwine

Welcome to this September issue of *African Health Sciences*, whose release we delayed, in order to meet the embargo (October 2022) on the international editorial on climate change. We joined that climate editorial, because of the importance of climate change challenges facing our village: planet earth!

## Other themes

The rest of this September 2022 issue has three main themes: sexual reproductive health,^1^–^12^ non communicable diseases,^13^–^42^ and infectious diseases^43^–^66^. The rest of the manuscripts are a cocktail of some other issues ranging from breastfeeding^67^, mortality of doctors in Uganda^68^, disaster preparedness^69^; radiographers in South Africa^70^; organophosphate occupational exposure^71^; hepatic arteries^72^; healthcare^73^; hand hygiene^74^; and iron status in Nigeria patients^75^.

The sexual reproductive health topics include erectile dysfunction among long distance drivers and *boda boda* riders^1^; coercion at sexual debut,^2^ fertility desire among people on antiretroviral therapy (ART),^3^ family planning among teachers,^4^ reproductive health among street youths,^5^ *Neisseria gonorrhea* in Uganda^6^, and syphilis in Tanzania.^7^ These are followed by papers on anemia in pregnancy in South Africa,^8^ male partner involvement in antenatal care in Kenya^9^; pregnancy termination in Uganda,^10^ drotaverine to shorten duration on labor,^11^ and anxiety and caesarean section.^12^

Then, somewhat surprising, we have 30 papers on non-communicable diseases.^13^–^42^ They include themes on cancer,^13^–^24^ diabetes mellitus,^25^–^29^ respiratory and cardiovascular illnesses,^30^–^35^ and others.^36^–^42^

The non-communicable diseases theme is then followed by infectious diseases, ranging from HIV,^43^–^49^ the COVID-19 pandemic,^50^–^57^ and others.^58^–^66^

We conclude the issue with a hybrid of papers^67^–^75^-*akatogo* of sorts!

Finally, we wish to thank our volunteers; editorial team and board; reviewers, authors, readers; and partners through AJPP and others, who, in one way or another, contributed to the sustainability and growth of AHS, as we continue our struggle to enhance publication of high-quality health science papers in Africa, the diaspora, and beyond. Thank you very much!
